# Intraoperative Findings of an Atypical Type II Endoleak from an Artery within the Aneurysmal Wall after Endovascular Aneurysm Repair

**DOI:** 10.3400/avd.cr.20-00109

**Published:** 2020-12-25

**Authors:** Baku Takahashi, Shinji Kamiya, Kengo Ohta, Yoshiharu Mori, Toshiyuki Yamada, Yosuke Nakai, Hisao Suda, Akira Mishima

**Affiliations:** 1 Department of Cardiovascular Surgery, Nagoya City University Hospital, Nagoya, Aichi, Japan; 2 Department of Radiology, Nagoya City University Hospital, Nagoya, Aichi, Japan

**Keywords:** endovascular aortic repair, atypical endoleak, vasa vasorum

## Abstract

A 75-year-old man underwent emergent endovascular aortic repair for a ruptured abdominal aortic aneurysm. Two years later, computed tomography revealed aneurysm enlargement with endoleaks. Next, late open conversion was performed. Intraoperatively, we detected a spurting type II endoleak from an artery within the aneurysmal wall, which was unconnected to any branch vessels outside the aneurysm, and surgical ligation and sacotomy was performed uneventfully. To our knowledge, this is the first report to intraoperatively identify a type II endoleak from an artery within the aneurysm wall. Even for atypical type II endoleak, such as this case, open surgical repair should be effective.

## Introduction

Type II endoleaks after endovascular aortic repair (EVAR) bleed into the aneurysm as retrograde flow from a branching vessel of the aneurysm. However, only two reports exist of the atypical type II endoleak not from an aortic branch, in which the vessel would be vasa vasorum, and both were subjected to endovascular treatment.^1,2)^ Herein, we performed late open conversion after EVAR and experienced not only typical type II endoleaks from the lumbar and median sacral artery but also atypical type II endoleak from an artery within the aneurysmal wall with no inflow blood vessels outside the aneurysm.

## Case Report

A 75-year-old man with a history of hypertension, diabetes, cerebral hemorrhage, and chronic kidney disease (creatinine 2.1 mg/dl) underwent emergent EVAR for a ruptured abdominal aortic aneurysm (maximum diameter 92 mm). The procedure utilized an Endurant® IIs bifurcated stent graft (Medtronic, Santa Rosa, CA, USA) and an Excluder® leg (W. L. Gore, Flagstaff, AZ, USA), with n-butyl-2-cyanoacrylate (NBCA) injected into the aneurysm to promote thrombosis. At the completion of the procedure, no endoleak was detected. A year later, computed tomography (CT) showed that the sac shrunk to 63 mm.

**Figure 1:**
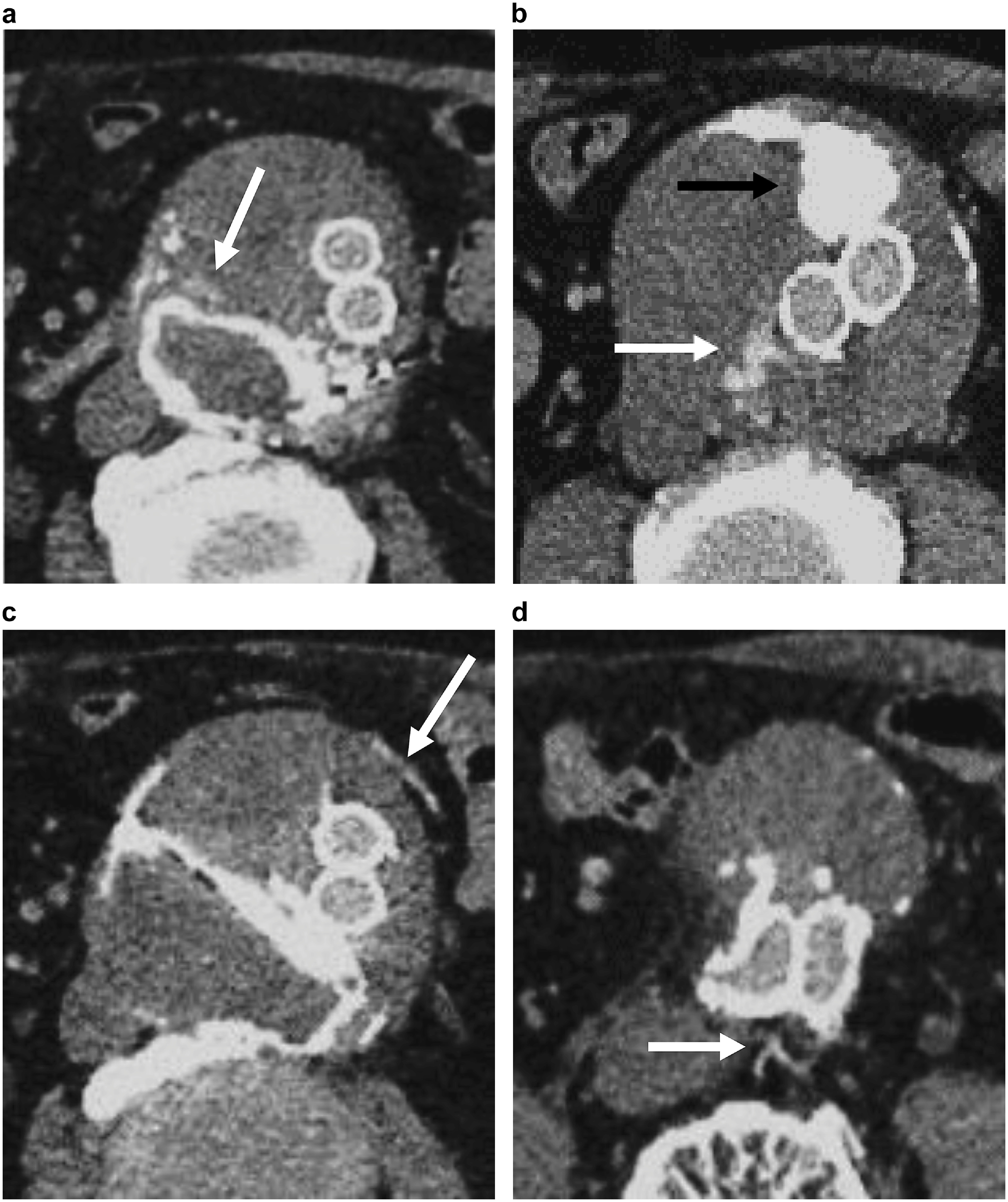
Fig. 1 Contrast-enhanced computed tomography findings 2 years after an endovascular aortic repair. Two endoleaks (white arrow) were observed: (**a**) the one with contrast medium leakage on the right side was not connected to any vessel and was not classified into a particular type and (**b**) the other one on the posterior side of the aneurysm originated from the third lumbar artery and was classified as type II. (**c**) The inferior mesenteric artery and (**d**) the median sacral artery were patent (white arrow). The black arrow indicates the n-butyl-2-cyanoacrylate in the sac.

However, 2 years after the EVAR, contrast-enhanced CT revealed that the aneurysm increased in size to 73 mm and that two endoleaks were present. One endoleak, in which the leakage of contrast medium was on the right side (**Fig. 1a**), was unconnected to any vessel and located away from the stent graft. The other was observed at the posterior side of the aneurysm, deriving from the third lumbar artery (**Fig. 1b**). The inferior mesenteric artery (IMA) and the median sacral artery were patent (**Figs. 1c** and **1d**). The patient provided informed consent for open surgery.

**Figure 2:**
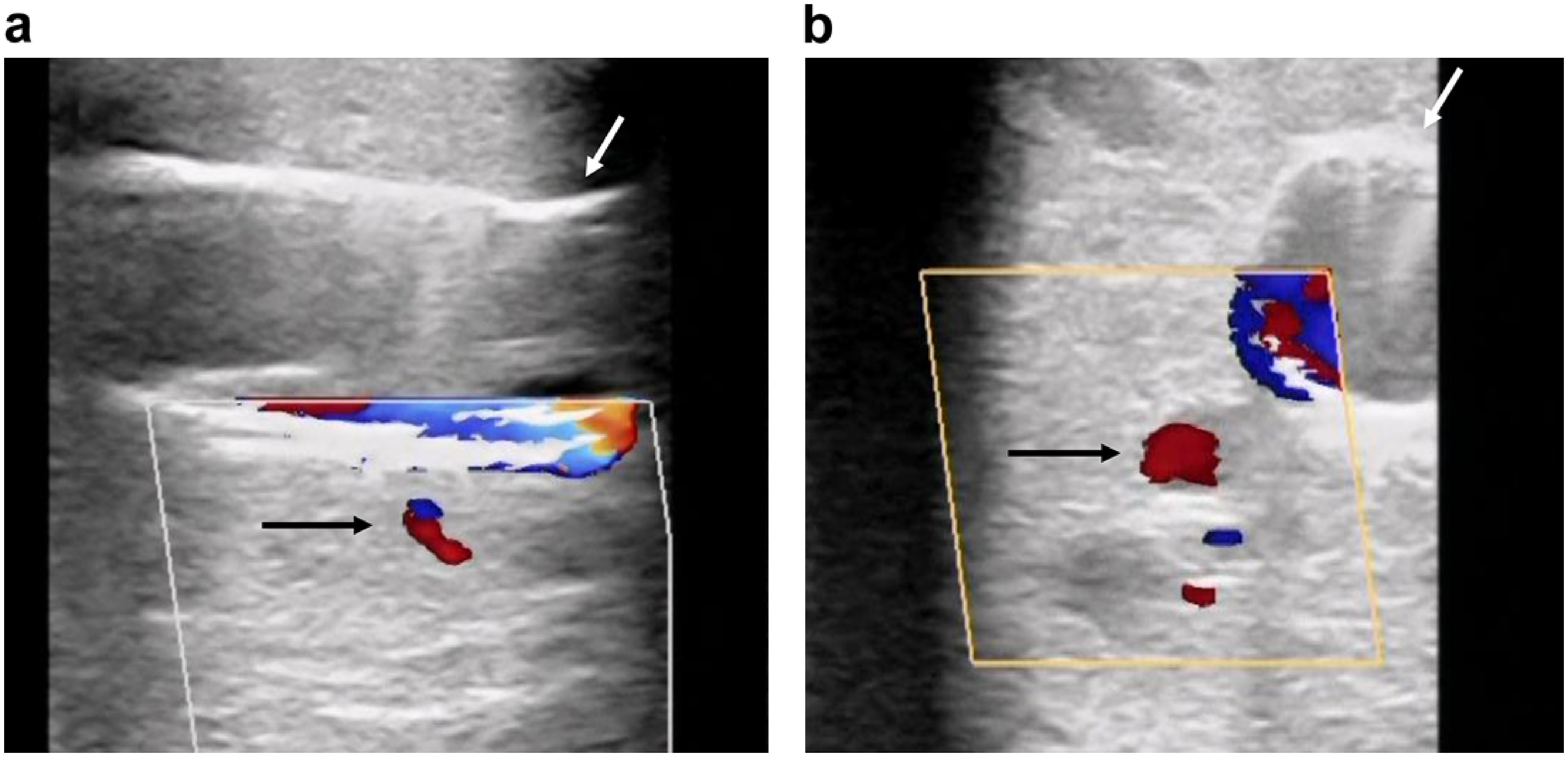
Fig. 2 Intraoperative epiaortic ultrasonography showing one of the target feeding vessels (black arrow) from the lumbar artery in the aneurysm next to the endograft (white arrow). (**a**) Long axis. (**b**) Short axis.

**Figure 3:**
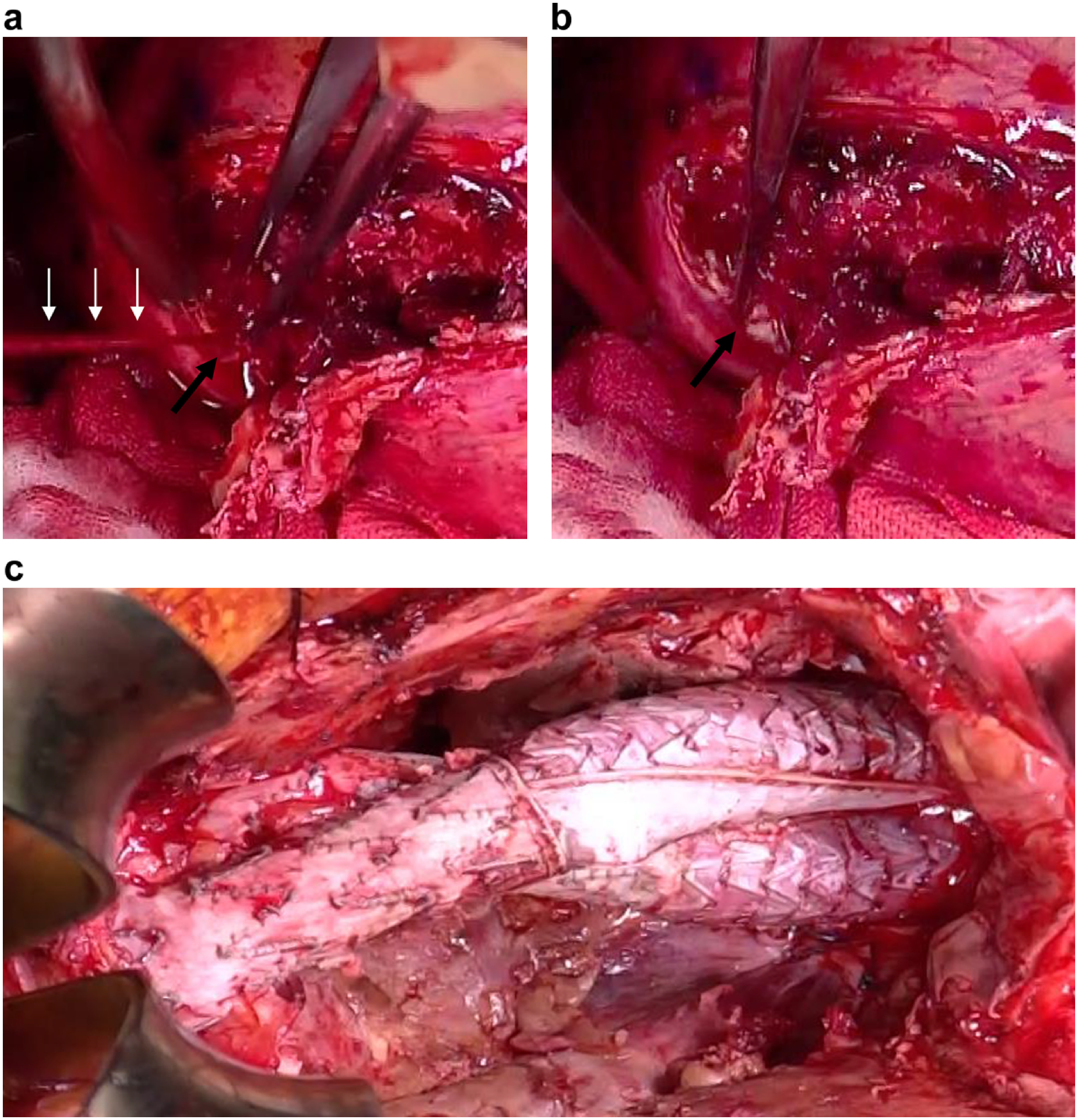
Fig. 3 Intraoperative findings. (**a**) Spurting bleeding (white arrows) from the aortic wall was observed on the right ventral side of the aorta. (**b**) The culprit vessel was an artery within the aneurysmal wall (black arrow). (**c**) No endograft damage occurred.

The abdominal aortic aneurysm was exposed via a transperitoneal approach. Intraoperative epiaortic ultrasonography showed that one of the feeding target vessels derived from a lumbar artery between the stent graft and the aortic wall (**Figs. 2a** and **2b**). When the sac was incised, blood filled the aneurysm, and the aneurysm kept opening laterally to the right even while the suspected bleeding point was pressed with a finger. Unexpectedly, spurting bleeding occurred into the sac from the aneurysm wall on the right ventral side of the aneurysm (**Fig. 3a**) from a pulsatile blood vessel just below the intima (**Fig. 3b**). The bleeding site was directly oversewn with 3-0 polypropylene suture, stopping the bleeding. No blood vessels leading to this vessel were found outside the aneurysm wall. The NBCA and thrombus in the aneurysm were removed. Also, bleeding occurred from a pair of lumbar arteries and the median sacral artery; these were directly sutured in a similar fashion. After confirming the IMA occluded and that no further bleeding or endograft damage was present (**Fig. 3c**), the remnant sac was closed carefully by sacotomy (Supplementary Video 1).

The postoperative course was uneventful, and postoperative follow-up contrast-enhanced CT showed no evidence of any further endoleak. The patient was followed up for one year, with no observed enlargement of the aneurysm.

## Discussion

Type II endoleaks reportedly occur after 10.2%–28.7% of EVARs.^3,4)^ After EVAR, the internal pressure of the aortic aneurysm decreases, and this can result in retrograde bleeding from the branch vessel into the aneurysm. The atypical endoleak here involved bleeding from the aneurysm wall into the sac, and thus was classified as type II endoleak; whereas in all previous reports of open repair for type II endoleaks, the bleeding derived from branch vessels. To the best of our knowledge, this is the first report of an atypical type II endoleak from an artery within the aneurysmal wall that was identified intraoperatively after EVAR, with no inflow blood vessels outside the aneurysm.

Two possible candidates exist for the identity of this atypical endoleak. The first is that it was part of the vasa vasorum, a network of vessels that feeds the major vessels. The vasa vasorum originates from the branch vessels of the aorta and is usually distributed within the adventitia,^5)^ although an EVAR can trigger substantial proliferation of the vasa vasorum in the aortic wall.^6)^ Two reports exist regarding the involvement of the vasa vasorum in aneurysmal enlargement as an atypical type II endoleak after EVAR, and both were treated with endovascular therapy.^1,2)^ To the naked eye, an endoleak from the vasa vasorum might appear to be from an artery within the aneurysmal wall, as in the present case. The second possible candidate vessel is that this was an anatomical variation, an abdominal aortic branch vessel running not outside but inside the aortic wall, following an intramural course. Although no report of such an anatomical variation exists, it is well established that the intramural artery runs through the wall of the ascending aorta as a congenital anomaly in the coronary arteries. In the present case, the artery may have been an abdominal aortic branch vessel such as the lumbar arteries or the gonadal artery courses within the wall of the aneurysm.

When to perform a second intervention for type II endoleaks remains controversial. In the present case, the second intervention was performed because the size of the aneurysm increased by 10 mm with type II endoleak in 1 year. Interestingly, the atypical type II endoleak was detected as a spurting hemorrhage suggestive of high flow during the surgery. This atypical endoleak may have involved antegrade flow, depending on the origin of the artery, consistent with the intraoperative findings. The reported probability of aneurysm rupture due to a type II endoleak after an EVAR is 0.9%, and more than one-third of these involved no sac expansion.^3)^ In such cases, the atypical antegrade flow of a type II endoleak may not be recognized; thus, a second intervention should be considered more often regardless of aneurysmal enlargement and time period.

Treatments for type II endoleaks include endovascular procedures, such as arterial or translumbar embolization and open surgical repair. For typical type II endoleaks such as those of the lumbar artery and IMA, surgical ligation of the causative blood vessel and sacotomy have been highly successful.^7)^ If the culprit blood vessel in the present case was an anatomical variation, this approach should be effective. Conversely, if the vessel was part of the vasa vasorum, whether this treatment would be the optimal management is unclear. In the reports of atypical type II endoleaks from vasa vasorum described earlier,^1,2)^ endovascular treatment with arterial embolization was performed; however, its effectiveness remains unknown because one case experienced recurrence, which was successfully treated with the translumbar direct sac embolization after failed arterial embolization.^2)^ In addition, the post-treatment condition of other patients was not described sufficiently.^1)^ Reports exist regarding the involvement of aneurysmal thrombus^6)^ or a hypoxic environment^8)^ in the development of vasa vasorum endoleaks after EVAR. Thus, open repair should be an efficient approach because it allows the removal of aneurysmal substances under oxygen.

## Conclusion

Type II endoleaks from an artery within the aneurysmal wall can occur after EVAR and may involve high flow. A second intervention should be considered more aggressively. Finally, open surgical repair should be effective for such atypical type II endoleaks.

## Editorial Policy

Informed consent was obtained from the patient.

## Supplementary Material

Type II endoleaks from an artery within the aneurysmal wall, lumbar artery, and median sacral artery were identified intraoperatively. The endoleaks were successfully ligated and sacotomy was performed.
